# Purification and Characterization of Bacteriocin Produced by *Weissella confusa* A3 of Dairy Origin

**DOI:** 10.1371/journal.pone.0140434

**Published:** 2015-10-16

**Authors:** Hweh Fen Goh, Koshy Philip

**Affiliations:** Microbiology Division, Institute of Biological Sciences, Faculty of Science, University of Malaya, 50603, Kuala Lumpur, Malaysia; Agricultural University of Athens, GREECE

## Abstract

A dramatic increase in bacterial resistance towards currently available antibiotics has raised worldwide concerns for public health. Therefore, antimicrobial peptides (AMPs) have emerged as a promisingly new group of therapeutic agents for managing infectious diseases. The present investigation focusses on the isolation and purification of a novel bacteriocin from an indigenous sample of cow milk and it’s mode of action. The bacteriocin was isolated from *Weissella confusa* A3 that was isolated from the sample and was shown to have inhibitory activity towards pathogenic bacteria namely *Bacillus cereus*, *Escherichia coli*, *Pseudomonas aeruginosa* and *Micrococcus luteus*. The bacteriocin was shown to be heat stable and functioned well at low pH (2 to 6). Reduction of activity was shown after treatment with proteinase K, trypsin and peptidase that confirmed the proteinaceous nature of the compound. MALDI-TOF analysis of the sample gave a mass approximating 2.7 kDa. The membrane of the bacteria was disrupted by the bacteriocin causing SYTOX^®^ green dye to enter the cell and bind to the bacterial DNA giving fluorescence signal. Bacterial cell treated with the bacteriocin also showed significant morphological changes under transmission electron microscope. No virulence and disease related genes can be detected from the genome of the strain.

## Introduction

Antimicrobial peptides (AMPs) are ubiquitous and natural antibiotics generated by a diverse range of microorganisms, plants, insect and mammalian cells. Recent attention has been drawn to AMPs as new antimicrobials to combat harmful microbes especially those resistant to conventional antibiotics. In the search for new antimicrobial agents, these may be used as templates for the design of novel drugs [1,2]. AMPs are divided into different groups based on their variable structural characteristics [3]. Those AMPs produced by bacteria to kill or inhibit other bacteria are known as bacteriocins [4,5].

In an effort to establish a new antimicrobial agent, attention was focused on lactic acid bacteria (LAB) in this study. This was chosen as some bacteriocins from LAB are well studied in the past and now used as safe and natural food preservatives. The best known example is nisin produced by *Lactococcus lactis* [6]. Except for a few species, LAB are considered as “Generally Recognized as Safe” (GRAS) and used in the production of fermented foods and beverages [7]. Some LAB produce antimicrobial compounds which may act as bacteriostatic or bactericidal agents. The antimicrobial activities are mainly due to the production of antimicrobial metabolites such as bacteriocins, hydrogen peroxide and organic acids [8].

The mechanisms of action of AMPs can generally be grouped into two classes. The first class is membrane disruptive following barrel stave, toroidal, carpet or micellar aggregate mechanisms. Second is the non-membrane disruptive class which targets the intracellular components [9]. In the first mechanism, bacteriocins selectively disrupt the cell membranes and the amphipathic structural arrangement of the bacteriocins is believed to play a significant role in this mechanism. Membrane disruption can also be caused by bacteriocins produced by milk-associated LAB. For example *L*. *lactis* strain which is usually found in milk is used as starter cultures in production of many fermented foods for producing safe biopreservatives [10]. Research reveals that milk consists of a wide range of bioactive compounds with antimicrobial, antihypertensive and antioxidant properties. Most of these bacteriocins are only released during milk fermentation implying the milk is presumed to generate these bioactive compounds [11].

The genus *Weissella* was reclassified in 1990. The species *Weissella confusa* isolated in the current study was formerly called *Lactobacillus confusus* and has been isolated from several sources. *Weissella confusa* is known to produce dextran which is an exopolysaccharide with α-glucans and a linear backbone made of α-(1→6)-linked d-glucopyranosyl units. *In situ* produced *W*. *confusa* dextran has been reported to be useful in improving the shelf-life, volume and nutritional value of bread [12–14]. Apart from the production of dextran, the bacteriocin produced by the genus *Weissella* received little attention. Until now only few bacteriocins known as weissellicin was identified from *Weissella* and report of bacteriocin produced by *Weissella confusa* is even rarer [15].

The aims of this study were to isolate and characterize a novel bacteriocin from LAB isolated from indigenous cow milk sampled in Malaysia and study its mode of action by using real-time PCR and SYTOX^®^ green dye that binds to nucleic acid of the affected target bacteria. Subsequently this disrupted the cell membrane. SYTOX^®^ Green dye has high binding affinity toward nucleic acid and easily penetrates those cells with compromised membranes but do not cross the intact membranes of live cells [16].

## Materials and Methods

### Isolation and Identification of Lactic Acid Bacteria from Fermented Milk

Sampling was done from raw cow milk randomly collected from two different restaurants (Al-Berkat Curry House and Grand City) located in Petaling Jaya, Selangor, Malaysia. The raw milk samples were pasteurized at 63°C for 30 minutes to kill the harmful bacteria present in the raw milk. The milk was left to ferment in a sterile flask under room temperature for two days. After fermentation process, LAB was isolated by growing on de-Mann, Rogosa and Sharpe (MRS) agar plates (Merck, Germany). These plates were incubated for 24 hours at 37°C. Single colonies were picked from the plates and sub-cultured two times to get a pure colony based on morphology. The isolates were grown in MRS broth for 24 hours and centrifuged at 10,000 x g for 20 minutes. The supernatant from each isolate was used for preliminary antimicrobial test by using agar well diffusion assay. 50 μl of the supernatant from each isolate was transferred to different wells on a Mueller-Hinton agar plate seeded with indicator bacteria. The bacteria isolate (A3) that produces potent antimicrobial activity was selected for further tests. For identification of bacteria, five tests were done including Gram-stain, spore stain, catalase test, oxidase test and the ability to grow on bile esculin azide agar (Merck, Germany). Cell morphology and Gram-stain were examined by light microscope at 1000X magnification. Schaeffer and Fulton’s spore staining method modified by [17] was followed. Bile esculin azide agar tested the ability of the bacteria to hydrolyze esculin in the presence of bile. Bacteria positive for esculin hydrolysis can hydrolyze the glycoside esculin to esculetin and dextrose. The bile in the agar was used to inhibit Gram-positive bacteria other than enterococci while the sodium azide inhibited Gram-negative bacteria. LAB was identified using API 50CHL (bioMérieux, France) which tested the ability of the bacteria to metabolize 49 kinds of carbohydrates. Pure colonies of the bacteria were transferred into the test medium and loaded into the test strip. The test strip was incubated for 48 hours at 37°C. The result obtained was examined for bacterial similarity to the database using the API software provided. The bacteriocin producer strain was identified by 16S rRNA gene sequencing using universal primers namely 27F (5'-AGAGTTTGATC(A/C)TGGCTCAG-3') and 1492R (5'-ACGG(C/T)TACCTTGTTACGACTT-3'). PCR reaction mixture (25 μl total) contains 2.5 μl PCR buffer, 2 μl of dNTP mix (2.5mM each), 1μl of each primer (20 pmol), 100ng of DNA template, 0.5 μl of i-Taq™ DNA polymerase 5U/μl supplied from iNtRON Biotechnology, Korea. PCR conditions used initial denaturation at 94°C for 5 minutes, denaturation at 94°C for 1 minute, annealing at 52°C for 1 minute, extension at 72°C for 1.5 minutes for 30 cycles with a final extension at 72°C for 10 minutes.

### Effect of Different Media on Bacteriocin Production

The bacteriocin producer (A3) was grown in 10 types of broth and tested against *B*. *cereus* to examine and compare the effectiveness of different culture media on the production of the bacteriocin. The media used was de Man, Rogosa and Sharpe (MRS), M17 supplemented with 1.5% glucose, M17 supplemented with 1.5% sucrose, M17 supplemented with 1.5% lactose, Brain-Heart Infusion (BHI), LAPTg [18], tryptic soy broth (TSB) with 1% Tween 80, Brucella broth, nutrient broth (NB) with 1.5% glucose and Miller's LB broth. After 18 hours incubation, the fermented broth was centrifuged at 10,000 x g for 20 minutes and the supernatant was precipitated with 80% ammonium sulphate overnight at 4°C. Then the pellet was centrifuged and dissolved in minimum amount of water and tested for inhibitory activity by well diffusion assay. Well diffusion assay was conducted by seeding *B*. *cereus* on Mueller- Hinton agar plate with sterile cotton swab and 5 mm diameter wells were made with a sterile cork borer. Then 50 μl of the crude bacteriocin purified from different media was transferred into separate wells. The plates were incubated at 37°C overnight and the inhibition zones were measured.

### Growth Curve and Production of Bacteriocin

The growth of the bacteriocin producer was measured using Miles and Misra method [19]. The growth of the bacteria was also monitored by measuring the optical density (O.D) at 600nm. Ten percent of starter culture of the bacteriocin producer at O.D 0.1 was added to 1 litre of sterile MRS broth. The colony forming unit (CFU) and the O.D. of the bacteria within 36 hours was monitored. The production of bacteriocin was monitored during log to stationary phases by measuring the inhibition zone of the crude bacteriocin. 20ml of the bacteria culture was centrifuged at 10,000 x g for 15 minutes and the supernatant was subjected to 80% ammonium sulphate precipitation. The dissolved precipitate from each interval was tested against *B*. *cereus* and the inhibition zone measured.

### Purification and Antimicrobial Assay of Bacteriocin

The test bacteria used were *Bacillus cereus* ATCC14579, *Escherichia coli* UT181, *Listeria monocytogenes* NCTC10890, *Pseudomonas aeruginosa* PA7, *Staphylococcus aureus* RF122, *Micrococcus luteus* ATCC10240, *Lactococcus lactis* A1 and *Enterococcus faecium* C1 from the culture collection maintained in the Microbial Biotechnology Laboratory, Microbiology Division of University of Malaya. All the test bacteria were maintained on Muller-Hinton agar (Merck, Germany) at 37°C. The concentration of the test bacteria was fixed at 0.1 value of O.D. at 600nm wavelength. The test bacteria were first lawn grown on Muller-Hinton agar (Merck, Germany) and wells were made using a cork borer. The test was carried out in triplicate. The plate was incubated overnight at 37°C and the inhibition zone was measured. For purification of the bacteriocin, the bacteria was grown in MRS broth for 18 hours and then centrifuged at 10,000 x g for 20 minutes. The bacterial pellet was discarded. The resulting cell-free supernatant was filtered with 0.2μm membrane filter (Sartorius, Germany). This supernatant was subjected to 80% ammonium sulphate precipitation to obtain the crude bacteriocin and then lyophilized. The lyophilized sample was then run through a column pre-packed with Amberlite XAD16 resin and eluted fractions then collected with increasing concentration gradients of acetonitrile and tested for antimicrobial activity using well diffusion assay. The active fraction from XAD column was further fractionated by size using Vivaspin (Sartorius, Germany) with different molecular weight cut off (MWCO) which is 0.2μm, 1,000,000 MWCO, 50,000 MWCO, 30,000 MWCO, 10,000 MWCO, 5,000 MWCO and 2,000 MWCO. Antimicrobial test was performed by spotting 20μl of the fraction on Mueller-Hinton agar seeded with *B cereus*. The active fraction collected from Vivaspin column was introduced into reverse-phase high-performance liquid chromatography (RP-HPLC) system (Waters, USA) using RP C18 column (Merck, Germany). The separation was carried out by gradient separation using two solvents: A (95% Mili-Q water (Millipore, USA) and 5% acetonitrile (Merck, Germany) and B (100% acetonitrile). The flow rate of the mobile phase was set at 1ml min^-1^. The following gradient was used: 0% solvent B for 3 minutes, 0–40% solvent B for 45 minutes followed by 40–100% solvent B for 5 minutes and then back to 100% solution A. Fractions were evaporated to remove acetonitrile and then tested for antimicrobial activity using *B*. *cereus* as a test indicator. Active fraction was collected at the same retention time during different HPLC runs and then pooled and lyophilized.

### Bacteriocin Activity Assay and the Effect of Various Concentrations of Bacteriocin on Optical Density of *B*. *cereus*

The minimum inhibitory concentration of the bacteriocin was performed using broth microdilution method proposed by Steinberg [20]. The indicator strains were inoculated into 5 ml of Mueller-Hinton broth and incubated at 18 h at 37°C on a shaker. Cultures were diluted with Mueller- Hinton broth to give 7 × 10^5^ CFU/ml. Two-fold serial dilutions were performed from the stock bacteriocin with Bradford concentration of 1.48 mg/ml in 0.2% BSA and 0.01% acetic acid in Eppendorf tubes (polypropylene). 11 μl of each concentration was added to a well in a 96-well poplypropylene plate. Then 100 μl of the diluted indicator strains was added into each corresponding well containing each concentration of the bacteriocin. The final concentrations of the bacteriocin were progressively halved to 74, 37, 18.5 until 1.16 μg/ml. The 96-well plate was incubated for 18 h at 37°C. MIC was taken as the lowest concentration of the bacteriocin that reduces growth by more than 50% compared to that of the control. 10 μl of the content of the well with higher MIC value was plated onto Mueller-Hinton agar and incubated at 37°C overnight. The lowest concentration of bacteriocin that prevents any viable cell growth was defined as the minimum bactericidal concentration (MBC). To determine the effect of various concentrations on the growth of *B*. *cereus*, the protocol of Skandamis [21] was followed with some modification. Briefly, overnight bacterial culture was diluted in Mueller-Hinton broth to give a final OD_615_ = 0.1. Then 100 μl of the bacterial suspension was added to a 96-well plate followed by 11 μl of the different bacteriocin concentrations as stated earlier. The final concentration of bacteriocin was similar to the concentration used to determine MIC. The plate was then read with Multiskan™ GO Microplate Spectrophotometer (Thermo Scientific, Finland) every 1 hour interval for a period of 24 hours.

### Stability Tests of Bacteriocin

The bacteriocin A3 was tested for stability in terms of inhibition on target microorganisms by varying its production in different carbon sources namely glucose, lactose and sucrose. This was repeated for thermal and pH stabilities. For accessing the thermal stability test, the bacteriocin was exposed to temperatures 40, 60, 80 and 100°C for 20 minutes. The heated bacteriocin was cooled to room temperature and tested for inhibition on *Bacillus cereus*. For the pH stability test, the bacteriocin was adjusted to a pH value ranging from 2 to 10 by using concentrated NaOH (Merck, Germany) and HCl (Merck, Germany). This was incubated for 2 hours at 25°C before determining the antimicrobial activity by agar well diffusion method. The vulnerability of the bacteriocin to breakdown by different enzymes namely proteinase K, lysozyme, lipase, catalase, lyticase, trypsin and peptidase sourced from Sigma- Aldrich was also tested and then checked for subsequent inhibition. 500 μl of the bacteriocin was treated with the enzymes with 1 mg/ml final concentration and a control without treatment was prepared. All preparations were incubated at 37°C for 1 hour before antimicrobial measurement with *B*. *cereus*.

### Molecular Weight Estimation with Sodium Dodecyl Sulfate-Polyacrylamide Gel Electrophoresis (SDS-PAGE) and MALDI-TOF

The molecular weight of the purified bacteriocin obtained from the HPLC fraction was estimated using Laemmli SDS-PAGE [22]. The gel used for the separation was 16.5% tris- tricine SDS-PAGE and the ladder used was Precision Plus Protein™ Dual Xtra Standards (Bio-Rad, USA). The gel was subjected to 100mV for one and a half hours and then stained with SimplyBlue™ SafeStain (Invitrogen, UK). The same sample also was subjected to matrix- assisted laser desorption ionization-time of flight mass spectrometry (MALDI-TOF MS). The targeted plate was spotted with aliquots consisting of 4μl of matrix and 4μl sample cleaned- up with zip-tip.

### Membrane Permeability Test

Real time PCR was used to monitor the quantity of DNA present when different concentrations of bacteriocin were added to the target bacteria. Bacteriocin shearing the membrane of the target bacteria can cause the bacterial DNA to be released and then bound with the fluorescent dye. Hence, higher fluorescence indicates the bacteriocin was more effective in terms of inhibitory activity on target bacteria. The membrane permeability test was done using SYTOX^®^ Green dye following the method used in previous studies with modification [23]. The indicator bacteria was grown until attaining an O.D value of 0.6 and then the bacterial pellets were washed with 10mM sodium phosphate buffer at pH 7.2 before centrifuging at 2,000 x g for 15 minutes. This step was repeated twice. The pellet was re-suspended in 5ml of 10mM sterile sodium phosphate buffer adjusted to pH 7.2. The bacteria was diluted again with sodium phosphate buffer and adjusted to an O.D value of 0.6 at 600nm wavelength. Then 5μl of SYTOX^®^ Green stock solution was added to 5ml of bacterial solution prepared as mentioned above. 90μl of the bacteria added with SYTOX^®^ Green was mixed with 10μl of bacteriocin. The positive control used was 1M sodium hydroxide and negative control contained only bacterial cells without bacteriocin. The experiment was performed in quadruplicate. The wells were sealed by using adhesive cover and then the plate was placed in the Step One Plus real-time PCR system (Applied Biosystem, USA).

### Effects of the Bacteriocin on Bacteria under Transmission Electron Microscope (TEM)

The test bacteria was treated with the bacteriocin and incubated for 3 hours at 37°C. Bacteria without addition of bacteriocin was prepared and used as a negative control. Then the bacteriocin was washed away thrice by using sodium phosphate buffer by centrifuging at 2,000 x g for 15 minutes. The cells were fixed with 4% glutaraldehyde and left overnight at 4°C. The samples were washed thrice with cacodylate buffer and post-fixed for 2 h with osmium tetroxide and cacodylate buffer in 1:1 ratio. Then it was washed thrice in cacodylate buffer and incubated in cacodylate buffer overnight. The samples were then washed thrice with water, once with uranyl acetate and again thrice with water. The samples were dehydrated in a graded ethanol series and embedded in Epon. Ultrathin sections (0.1 μm) were prepared and coated on copper grids and stained with uranyl acetate and lead citrate. The grids were examined using LEO-Libra 120 transmission electron microscope (Carl Zeiss, Germany).

### Basic Analysis of Genome Sequence

The total genomic DNA of the bacteria was extracted with DNeasy Blood & Tissue Kits (Qiagen, Valencia) following manufacturer’s instruction. The DNA was fragmented into a size ranging 600–900bp using the Covaris S220 system (Applied Biosystems, USA). DNA library (using 800ng sonic cleaned DNA) was prepared by Nextflex DNA Sample Prep Kit (Bio scientific, Texas, USA) complying with the manufacturer’s instructions. Library QC was performed on Bioanalyzer (Agilent Technologies) to check the fragment size distribution. The QC benchmarked library was sequenced on MiSeq sequencer (Illumina) with MiSeq Reagent Kits v3 (300 cycles, paired-end). Fluorescent images were analyzed using the MiSeq Control Software and FASTQ-formatted sequence data was created using MiSeq Reporter Analysis. The genome sequence was analyzed by Rapid Annotation Subsystem Technology (RAST) server (http://rast.nmpdr.org/).

## Results

### Isolation and Identification of Lactic Acid Bacteria from Fermented Milk

In the preliminary antimicrobial test, one LAB was showed to give inhibitory activity against the test bacteria. The bacterial colony morphology on MRS agar plate showed as medium sized and milky-white in colour, circular, convex and elevated with entire margins. The isolate was negative for catalase and oxidase and did not produce spores based on staining with malachite green. The bacteria appeared rod shaped under light microscope. Bile esculin test showed that it was able to weakly hydrolyze esculin in the presence of bile. Based on the interpretation of the API database, the bacteria colony was shown to be 99.9% similar to *Weissella confusa*. Genomic analysis 16S rRNA gene sequencing confirmed the isolate was *Weissella confusa*. The sequence obtained from 16S rRNA gene sequencing was deposited in the NCBI gene bank database under accession number KJ476186 and was given the strain designation A3.

### Effect of Different Media on the Production of Bacteriocin

Several commercially media available were tested for supporting the growth and production of bacteriocin. Among the 10 media used, MRS gave the highest activity followed by LAPTg and M17 supplemented with different carbohydrates. The other 5 media (BHI, TSB with 1% tween 80, Brucella broth, NB with 1.5% glucose and Miller's LB broth) also can be used to grow the bacteria but the production of bacteriocin was lower and no bacteriocin could be recovered from the culture grown in Brucella broth. The inhibition zone is shown in Table 1.

**Table 1:** Antimicrobial activity of bacteriocin recovered from *W*. *confusa* cultured in different media against *Bacillus cereus* ATCC14579.

Media	MRS	M17+1.5% Sucrose	M17+1.5% Glucose	M17+1.5% Lactose	BHI	LAPTg	TSB+1% tween 80	NB+1.5% glucose	LB+1.5% glucose	Brucella broth
**Inhibition zone** *	11.62	9.68	9.22	7.88	7.02	9.52	6.84	6.18	7.86	-

* Inhibition zone measures in millimeter (mm)

### Growth Curve and Production of Bacteriocin Study

**Fig 1:**
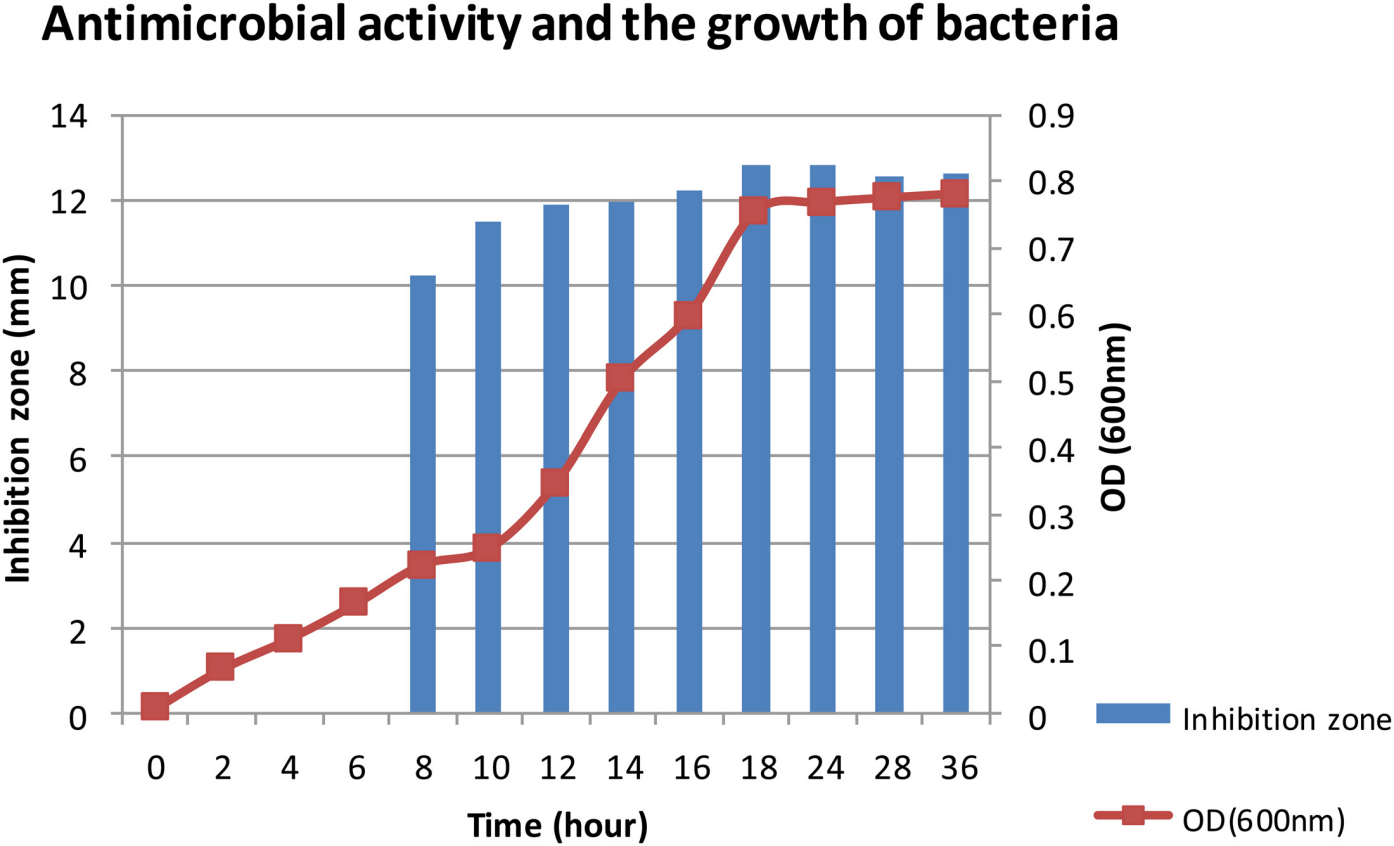
Antimicrobial activity and the growth of the bacteria. The antimicrobial activity started to be detected at 8 hours of incubation period and reached optimum at 18 hours of incubation period.

The growth curves (Fig 1) the bacteria reached stationary phase after 18 hours of incubation. According to the inhibition zone from the crude bacteriocin purified from the supernatant, the bacteriocin started to produce at 8 hours of incubation and reached optimum production at 18 hours incubation period. The antimicrobial activity remained almost the same between 18 to 24 hours of growth. However the activity of bacteriocin started to decrease at 28 hours of growth (Fig 1). Therefore the bacteriocin was best harvested between 18 to 24 hours to get optimum production.

### Purification and Antimicrobial Assay of Bacteriocin

**Fig 2:**
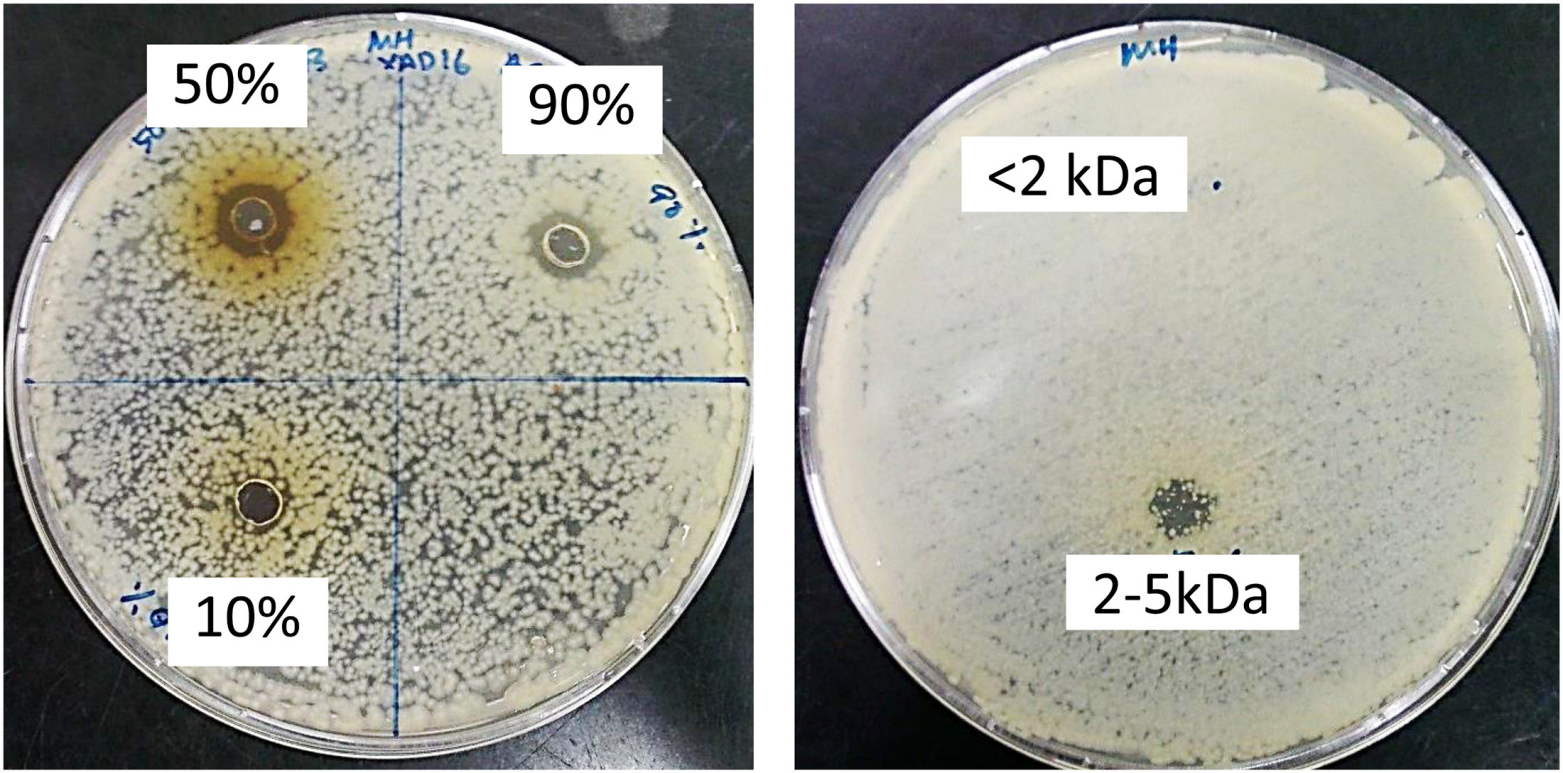
Antimicrobial assay. Antimicrobial activity of different fractions from Amberlite XAD 16 column (a) and two different fractions (< 2kDa and 2–5kDa) from Vivaspin (b).

**Fig 3:**
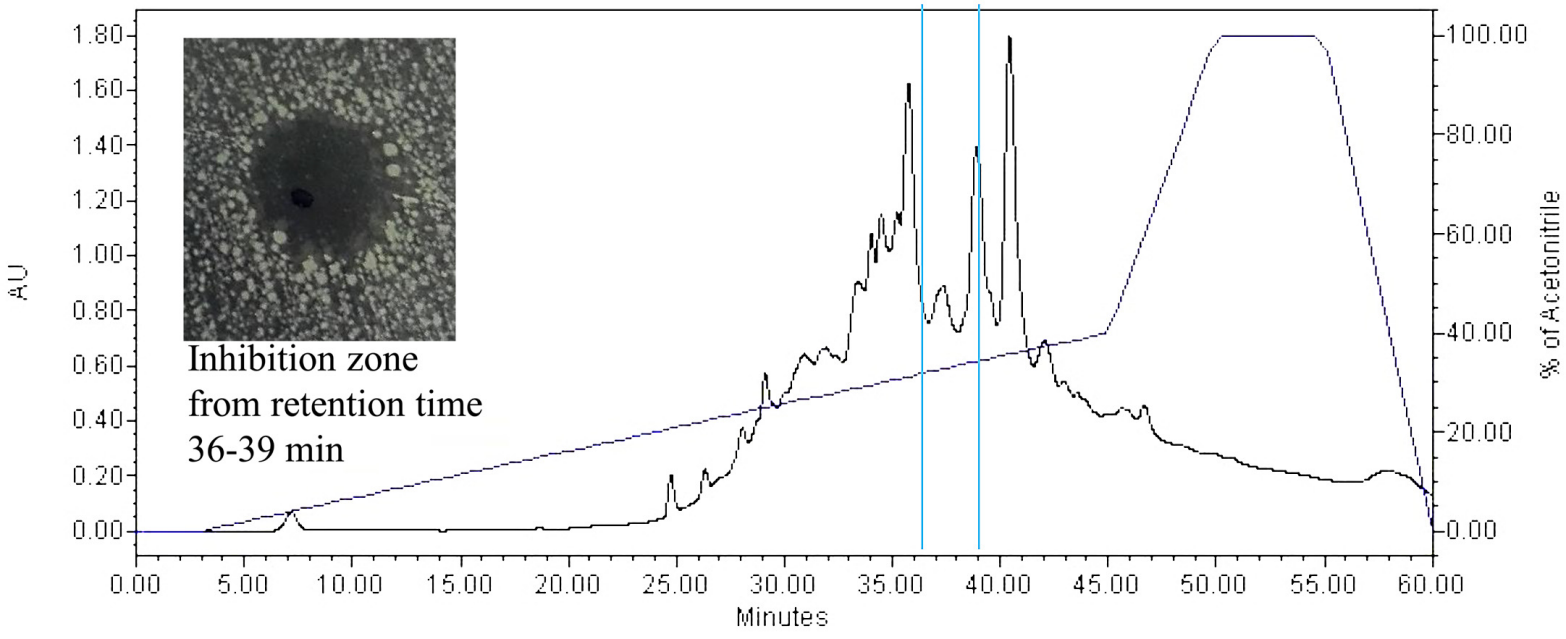
RP-HPLC Profile. RP-HPLC profile of active faction isolated from *Weissella confusa* with antimicrobial activity detected during 36 to 39 minute elution period.

The bacteriocin produced by the *W*. *confusa* showed inhibition against all test bacteria except *L*. *monocytogenes* NCTC10890 and *S*. *aureus* RF122. The inhibition zone of the ammonium sulphate precipitated extract against different test bacteria is shown in Table 2. The crude bacteriocin was desalted with XAD 16 column to remove the ammonium sulphate. Three fractions were obtained from the Amberlite XAD 16 column. Only fractions eluted out with 50% and 90% of acetonitrile gave activity against *B*. *cereus* (Fig 2a). The active fractions were combined and fractionated with Vivaspin column. The sample fraction of size 2–5kDa showed positive result when tested against *B*. *cereus* (Fig 2b). The bacteriocin from Vivaspin was injected into HPLC and the run under the set gradient for 1 hour (Fig 3). The active bacteriocin was eluted out from 36 to 39 min and inhibited *B*. *cereus*.

**Table 2:** Inhibition zones and MIC value of the crude and purified bacteriocin against test bacteria strains.

Test bacteria	Ammonium sulphate precipitation*	Amberlite XAD 16*	MIC for HPLC fraction (μg/ml)	MBC for HPLC fraction (μg/ml)
*Bacillus cereus* ATCC14579	10.67±0.58	11.89±0.06	9.25	37
*Escherichia coli* UT181	7.98±0.06	8.49±0.25	18.5	74
*Listeria monocytogenes* NCTC10890	0	0	-	-
*Pseudomonas aeruginosa* PA7	11.83±0.89	11.59±0.26	18.5	74
*Staphylococcus aureus* RF122	0	0	-	-
*Micrococcus luteus* ATCC10240	8.59±0.03	11.57±0.10	9.25	37
*Lactococcus lactis* A1	8.72±0.52	8.93±0.10	37	>74
*Enterococcus faecium* C1	9.25±0.10	9.59±0.28	37	>74

*Inhibition zones measured in ±standard deviation millimeter (mm).

### Bacteriocin Activity Assay and the Effect of Various Concentrations of Bacteriocin on Optical Density of *B*. *cereus*

**Fig 4:**
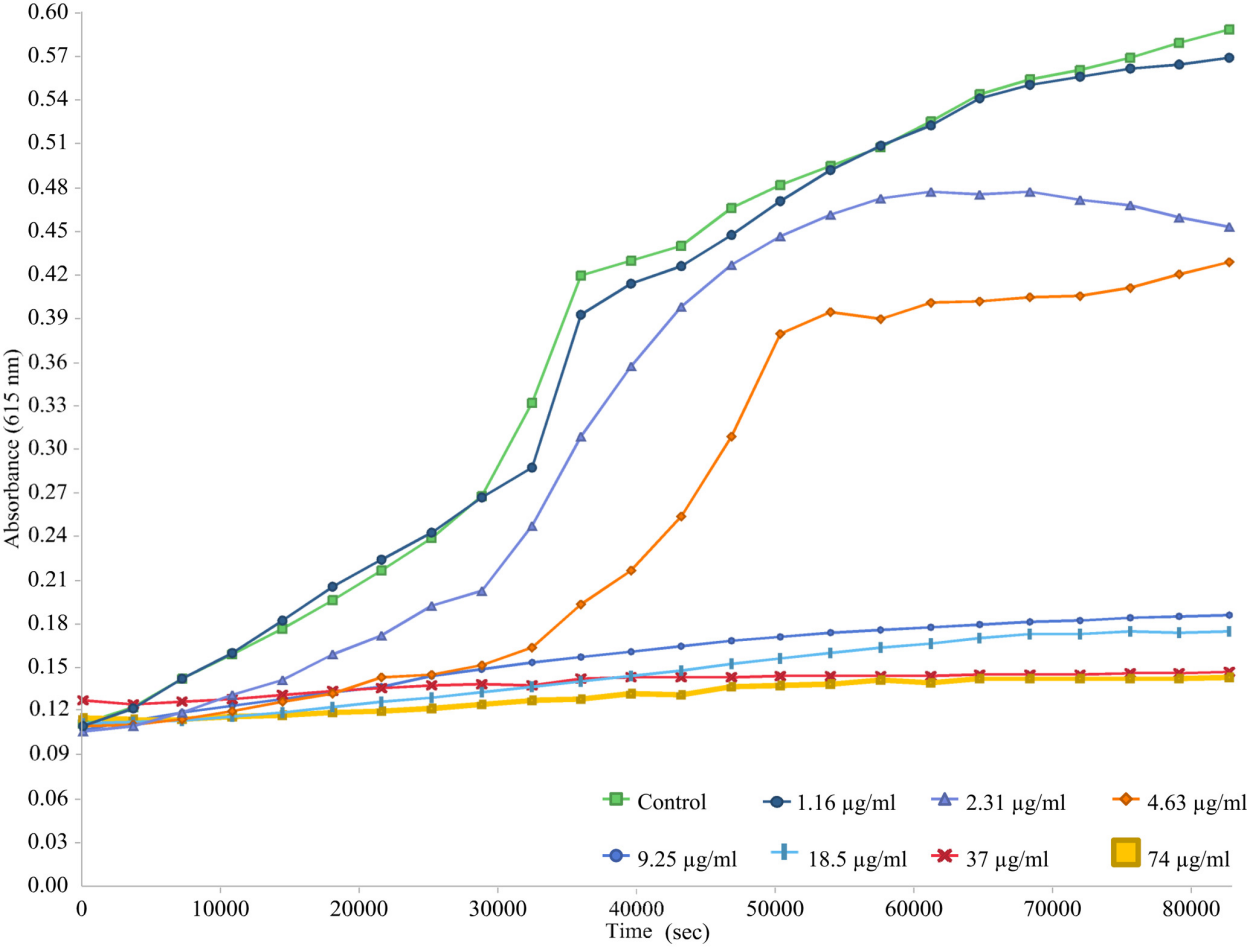
Effect of various concentration of bacteriocin on the growth of *B*. *cereus* at 37°C. The optical density was measured at 615 nm every hour continuously for a period of 24 hours.

The MIC value (Table 2) of the bacteriocin was 9.25 μg/ml against both *B*.*cereus* and *M*. *luteus*. The MIC value increased to 18.5 μg/ml against *E*.*coli* and *P*. *aeruginosa*. A higher MIC of 37 μg/ml was required to inhibit *L*. *lactis* and *E*. *faecium*. After plating 10 μl of the overnight bacteriocin with the bacterial solution from the wells but without any bacterial growth after incubation, the MBC obtained for *B*.*cereus* and *M*. *luteus* was 37 μg/ml. The MBC for *E*.*coli* and *P*. *aeruginosa* was 74 μg/ml whereas *L*. *lactis* and *E*. *faecium* required a concentration exceeding 74 μg/ml to produce bactericidal effect. The addition of different concentrations of bacteriocin affected the growth of *B*. *cereus* (Fig 4). The growth of bacteria can be observed in concentrations below 4.63 μg/ml. The growth of *B*. *cereus* was extremely slow in concentrations of 9.25 and 18.5 μg/ml. The optical density of *B*. *cereus* remained almost unchanged in concentrations of bacteriocin exceeding 37 μg/ml.

### Stability Test of Bacteriocin

The effect of the three different carbohydrates as carbon source supplemented in MRS showed that there was no significant contribution of the different carbohydrates on the inhibition. But 2% of glucose gave slightly better inhibition zones compared to sucrose and lactose. The heat stability test showed that the bacteriocin was heat stable and still showed inhibitory activity after exposing to 100°C for 30 minutes. The bacteriocin functioned well at a low pH of 2 to 6. The antimicrobial activity decreased upon exposure to high pH. For enzyme stability test, the bacteriocin was stable after treatment with lysozyme, lipase, catalase and lyticase but showed reduction of the activity after treatment with proteinase K, trypsin and peptidase. The reduction of activity by proteolytic enzymes confirmed that the antimicrobial substance was not of lipid nature but was proteinaceous in nature. The results of heat, pH and enzyme stability tests are summarized in Table 3.

**Table 3:** Heat, pH and Enzyme stability tests of the bacteriocin against *Bacillus cereus* ATCC14579.

Stability test	Inhibition zone (mm)	Residue activity (%)^¥^
**Heat**		
Untreated control	11.64	100
40°C	11.42	96.69
60°C	11.14	92.47
80°C	11.12	92.17
100°C	11.00	90.36
**pH**		
Untreated control	11.40	100
2–4	11.56–11.58	96.25–102.81
5–6	9.54–10.82	70.94–90.94
7–10	8.44	0
**Enzyme (1 mg/ml)**		
Untreated control	11.50	100
Proteinase K	8.78	58.12
Lysozyme	11.48	99.69
Lipase	11.44	99.08
Catalase	11.50	100
Peptidase	8.86	59.38

^**¥**^ $Residue\;activity\;=\;\frac{\left(Inhibition\;zone-5\right)}{\left(Inhibition\;zone\;of\;Untreated-5\right)}\times 100$

### Molecular Weight Estimation with Sodium Dodecyl Sulfate-Polyacrylamide Gel Electrophoresis (SDS-PAGE) and MALDI-TOF

**Fig 5:**
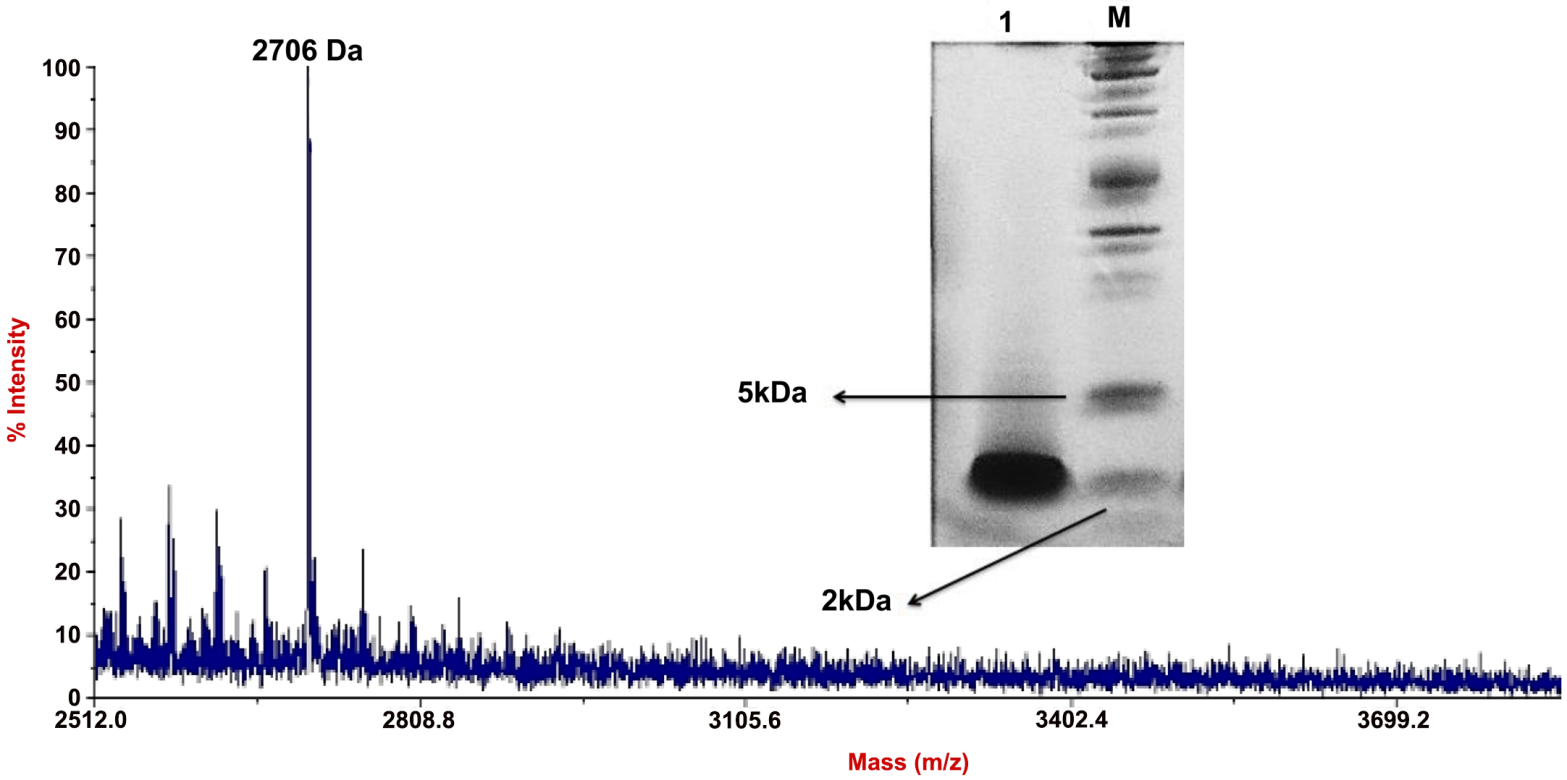
MALDI-TOF mass spectrometry and SDS-PAGE of purified bacteriocin. MALDI-TOF MS analysis of the HPLC fraction and SDS-PAGE gel picture. Lane 1 is fraction from HPLC, Lane M is Precision Plus Protein™ Dual Xtra Standards (Bio-Rad, USA).

When the HPLC sample was subjected to SDS-PAGE, only a single band was detected confirming its high purity. The purified bacteriocin showed molecular weight approximately 2.5 kDa compared to the marker used (Fig 5). The MALDI-TOF chromatogram (Fig 5) showed that the sample had a high density peak at 2706.6855Da. Therefore the possible molecular weight of the sample was approximately 2.7 kDa.

### Membrane Permeability Test

**Fig 6:**
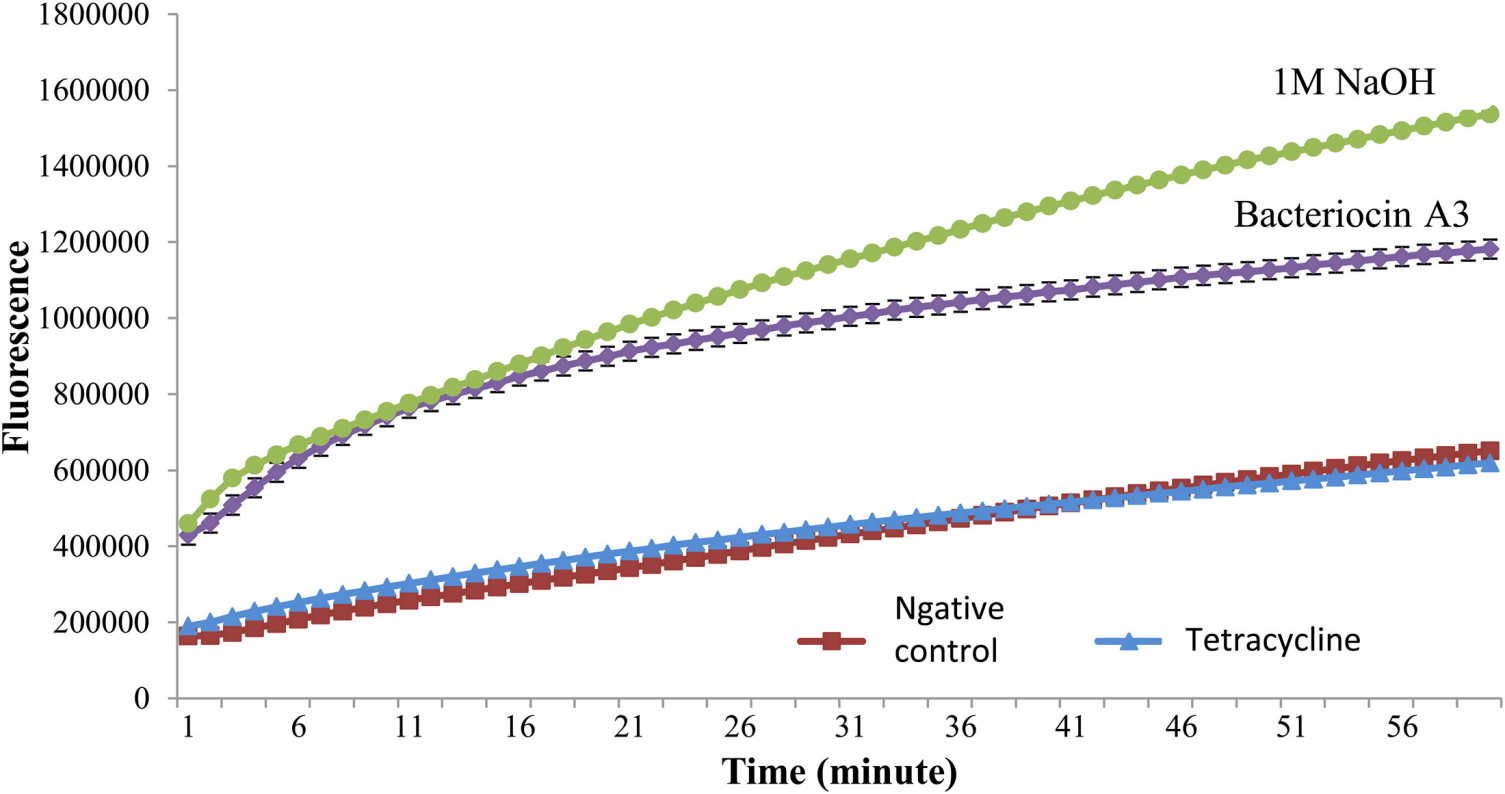
Membrane permeability test. Real time PCR fluorescence pattern of bacteriocin from *W*. *confusa*, negative control, positive control (NaOH, 1M) and tetracycline.

The real time PCR fluorescence (Fig 6) showed that the bacteria treated with bacteriocin from *W*. *confusa* had highest fluorescence density after positive control (NaOH, 1M). Negative controls without adding any bacteriocin and with tetracycline added showed the lowest fluorescence. This result proved membrane disruption occur when bacteriocin from *W*. *confusa* was added into the test bacteria allowed the fluorescence dye to bind to the nucleic acid of the test bacteria and hence giving high fluorescence intensity.

### Effects of the Bacteriocin on Bacteria under Transmission Electron Microscope (TEM)

**Fig 7:**
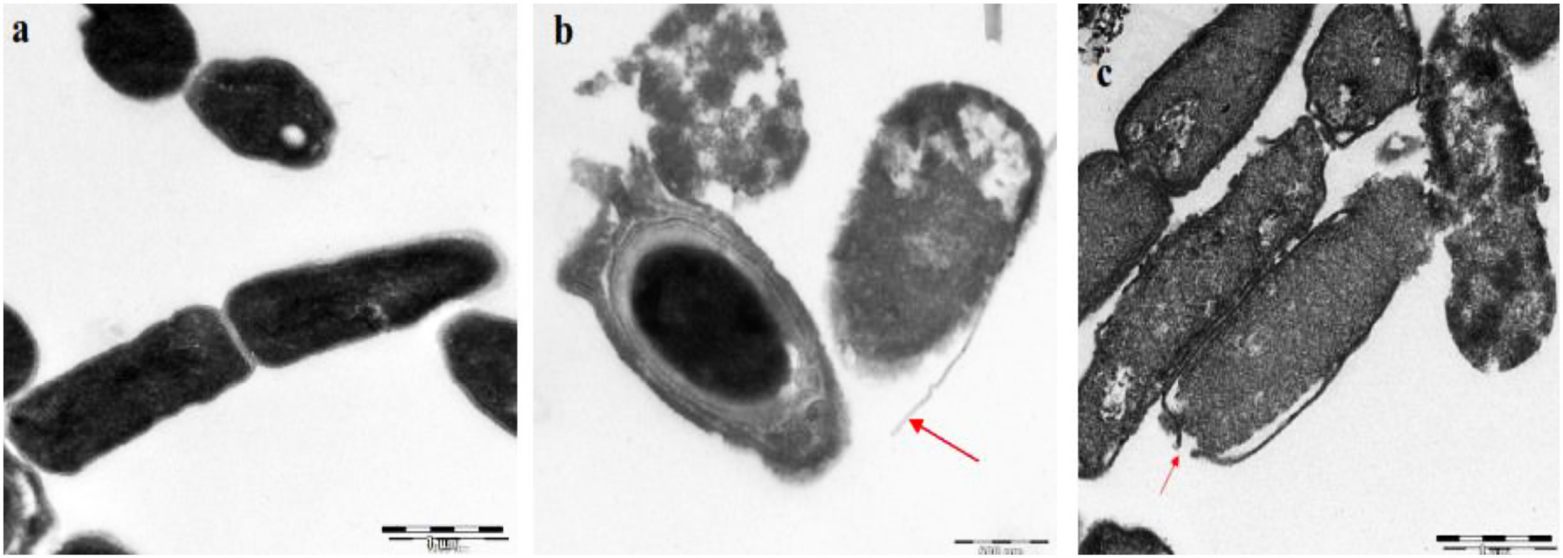
Bactericidal effect of Bacteriocin A3 on *B*. *cereus*. TEM images of *B*. *cereus* cells before (a) and after (b & c) treatment with bacteriocin extracted from *W*. *confusa*. Bar indicates 1μm for (a & c) and 500nm for (b). Arrows indicate destruction of membrane.

*Bacillus cereus* treated with bacteriocin and untreated control was observed under transmission electron microscope. The untreated cell showed complete membrane (Fig 7a) and the bacterial cells treated with bacteriocin shows disruption on the membrane. In Fig 7b, the bacterial cell membrane was sloughed off. Fig 7c showed a pore formation on the cell membrane.

### Basic Analysis of Genome Sequence

**Fig 8:**
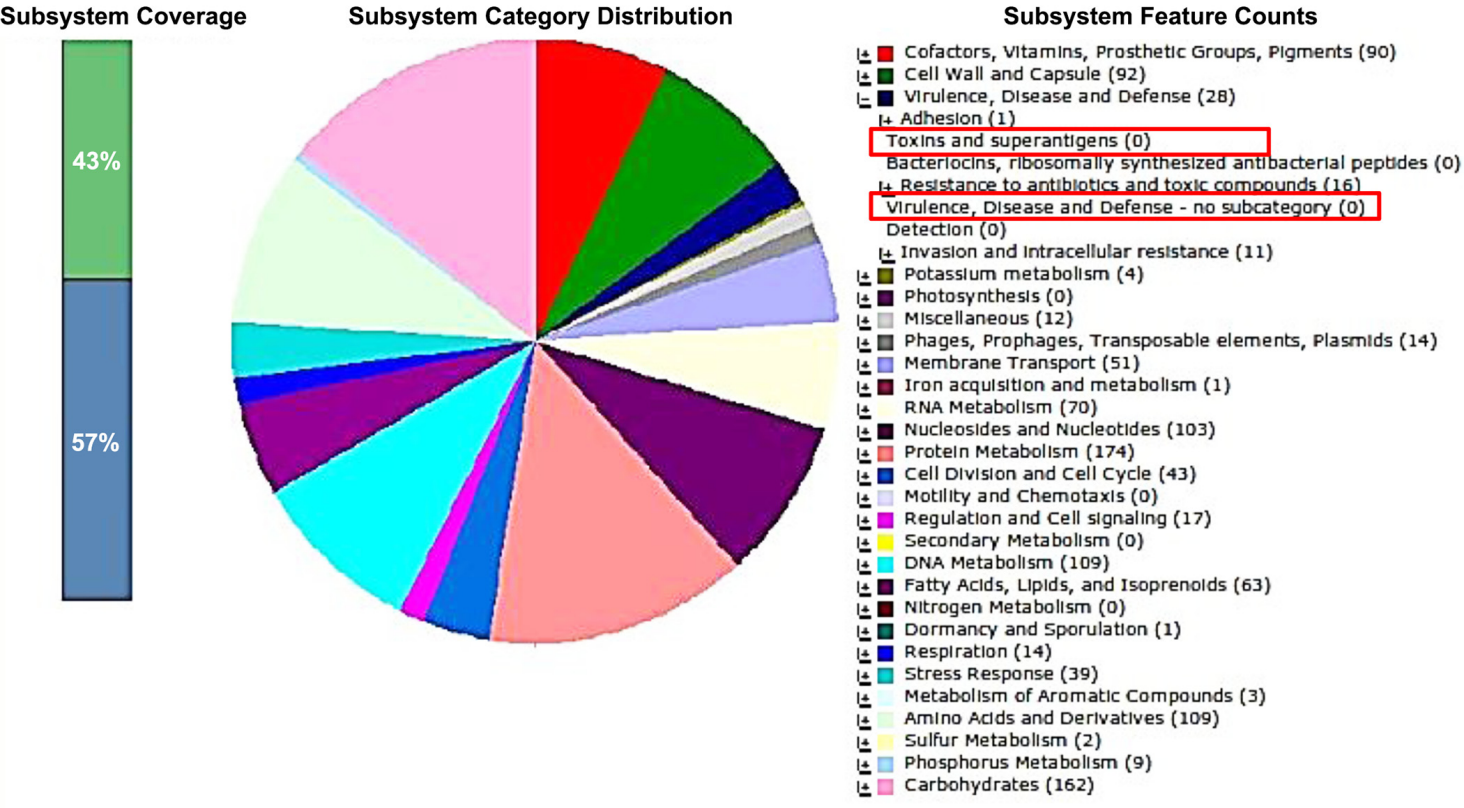
Subsystem feature of the genome sequence of *W*. *confusa* A3 analysed with RAST server. The red box indicated absence of genes in toxins and superantigens and virulence, disease and defense genes.

The bacteria genome was analyzed with RAST server for virulence, disease and defense subsystems. It was conclusively observed that the *W*. *confusa* strain used in our study did not harbour any genes related to toxins and production of superantigens. Virulence, disease and defense genes were also not detected from the genome of the bacteria (Fig 8). For gene encoding bacteriocin production, possible colicin V production protein was detected from the genome. The gene encoding possible colicin V production was translated into amino acid sequence and searched with UniProt (http://www.uniprot.org/blast/) showing the amino acid sequence to be 100% identical to colicin V from *W*. *confusa* LBAE C39-2. It can therefore be established that the bacteria is safe for use in the food industry.

## Discussion

*Weissella confusa* can be isolated from sugarcane, carrot juice and fermented food and occasionally from raw milk, saliva, sewage and clinical samples. They also form part of the normal microbiota in human intestine (Schleifer, 2009). Before fermenting the milk in this study, it was pasteurized to eliminate pathogenic and food spoilage bacteria while leaving the good bacteria intact which can withstand high temperature. These good bacteria comprise mostly of the LAB. During fermentation, the bacteria utilized the nutrients in the milk to multiply. Hence, this step was also known as the enrichment step. *W*. *confusa* can hydrolyze esculin in the presence of bile. It hydrolyzed the glycoside esculin to form dextrose and esculetin which react with ferric citrate producing a dark brown or black phenolic iron complex. This test is commonly used to presumptively identify Group D Streptococci but also showed positive reaction for *W*. *confusa* in our study. Bacteriocin production is always influenced by the carbon and nitrogen sources of the growth media. The maximum bacteriocin production of *W*. *confusa* A3 was obtained from MRS media. In the past studies, MRS media was used to cultivate LAB since it was rich in organic carbon and nitrogen sources which enhanced the growth and production of bacteriocin.

*W*. *confusa* can be isolated from sugarcane, carrot juice, fermented food and occasionally from raw milk, saliva, sewage and clinical samples. They also form part of the normal microbiota in human intestine [24]. Before fermenting the milk in this study, it was pasteurized to eliminate pathogenic and food spoilage bacteria while leaving the good bacteria intact which can withstand high temperature. These good bacteria comprise mostly of the LAB. During fermentation, the bacteria utilized the nutrients in the milk to multiply. Hence, this step was also known as the enrichment step. *W*. *confusa* can hydrolyze esculin in the presence of bile. It hydrolyzed the glycoside esculin to form dextrose and esculetin which react with ferric citrate producing a dark brown or black phenolic iron complex. This test is commonly used to presumptively identify Group D Streptococci but also showed positive reaction for *W*. *confusa* in our study. Bacteriocin production is always influenced by the carbon and nitrogen sources of the growth media. The maximum production of bacteriocin from *W*. *confusa* A3 was obtained with MRS media. In past studies, MRS media was used to cultivate LAB since it was rich in organic carbon and nitrogen sources which enhanced the growth and production of bacteriocin [25–27].

The peptides or bacteriocins produced by other genera of LAB such as *Lactobacillus*, *Enterococcus*, *Leuconostoc*, *Streptococcus*, and *Carnobacterium* were reported in previous studies. Nevertheless, there was insufficient studies on bacteriocins from *Weissella* sp especially *W*. *confusa* [28]. The bacteriocins isolated from *W*. *confusa* was found to belong to class II bacteriocins from past research because the antibacterial activity was stable after exposing to low pH, pepsin, proteinase or heat [29]. In this study the antibacterial activity of the bacteriocin also showed similar characteristics.

The bacteriocin extracted from the bacteria in the current study showed good inhibitory activity against *B*. *cereus*, *E*. *coli*, *P*. *aeruginosa*, *M*. *luteus*, *L*. *lactis* and *E*. *faecium*. This indicates that the bacteriocin produced by *W*. *confusa* has a broad spectrum of antimicrobial activity inhibiting both Gram-positive and Gram-negative bacteria used in this study.

The optimal bacteriocin production of the bacteria was obtained at 18 hours of incubation at 37°C that marked the beginning of the stationary growth phase of the bacteria followed by decrease of production during the rest of the stationary phase. Similar trend of bacteriocin production was reported in previous studies for nisin and other bacteriocins [30,31]. In this study, 4 steps of the purification method were carried out. Firstly, we extracted the total protein and peptide from the supernatant of the producer strain by ammonium sulphate precipitation. Then, in order to remove the ammonium sulphate residue from the extracted bacteriocin and at the same time remove hydrophilic protein, Amberlite XAD 16 column was used. This is based on a similar method used with a different type of Amberlite XAD column to purify lantibiotic from *Paenibacillus polymyxa* strain [32]. The third step of purification involved the use of Vivaspin centrifugal column to separate the peptides or proteins in the sample into two portions based on their molecular weights. Then the fraction from Vivaspin was further fractionated with RP-HPLC using C18 column. Vivaspin results in the current study also suggested that the bacteriocin was of smaller size ranging from 2 to 5 kDa. This was further confirmed by MALDI-TOF result (Fig 5) indicating the size was approximately 2.7 kDa. The size was different from weissellin-A which was a 4450 Da class IIa bacteriocin purified from *Weissella paramesenteroides* DX [33] and weissellicin 110 which was of 3,487.8 Da size purified from *Weissella cibaria* 110 [28]. Bacteriocin with similar molecular weights reported from past studies included pediocin A from *Pediococcus acidilactici* [34] and bacteriocin ST33LD from *Leuconostoc mesenteroides subsp*. *mesenteroides* [35].

The mode of action study using real time PCR showed tetracycline used gave low fluorescence while the bacteriocin from *W*. *confusa* gave higher fluorescence. This was attributed to the disruption of the bacterial membrane or lack of it. When no DNA is released from the cell there is reduced fluorescence. The fluorescence dye only binds to the inner DNA of the bacteria in the event of membrane disruption by the bacteriocin. So once the bacteriocin disrupts the cell membrane, the SYTOX^®^ Green binds to the inner DNA and fluorescence is then detected by real time PCR. This evidence indicated that the bacteriocin disrupted the cell membrane. SYTOX^®^ Green appeared to be a reliable molecular probe to detect membrane disruption of targeted sensitive bacterial strains treated with bacteriocins [23]. The mechanism of action of tetracycline is not by membrane permeabilization but by inhibition of protein synthesis. Hence, low fluorescence was detected in the treatment with tetracycline.

The electron micrograph of the untreated bacterial cells had smooth membranes and uniform shapes but the treated bacterial cells in our study became shrunken after treatment indicating water and ion loss from the cell. After treatment the membrane sloughed off (Fig 7b & 7c) but retained the cytoplasmic content of the cell. Besides, spore formation could also be seen after bacteriocin treatment. *Bacillus cereus* formed spores under unfavourable external conditions. This indicated that the bacteriocin added stress to the *Bacillus cereus* causing it to form spores to survive harsh conditions.

The strain also proved to be non-virulent and does not produce toxins and superantigens based on genome analysis from this investigation. This novel bacteriocin isolated from an indigenous milk source has potentials as an antimicrobial in the food industry. Further work is being pursued to facilitate its development as a food preservative or as a new antibiotic.
